# Influence of *Cmr1* in the Regulation of Antioxidant Function Melanin Biosynthesis in *Aureobasidium pullulans*

**DOI:** 10.3390/foods12112135

**Published:** 2023-05-25

**Authors:** Wan Wang, Kai Zhang, Congyu Lin, Shanshan Zhao, Jiaqi Guan, Wei Zhou, Xin Ru, Hua Cong, Qian Yang

**Affiliations:** 1 School of Life Science and Technology, Harbin Institute of Technology, Harbin 150006, China; 17862702273@163.com (W.W.); zhangkaiown@163.com (K.Z.); g13610013008@163.com (J.G.); zw3268133440@163.com (W.Z.); 18745180137@163.com (X.R.); conghua@hit.edu.cn (H.C.); 2 National Engineering Research Center of Cereal Fermentation and Food Biomanufacturing, School of Food Science and Technology, Jiangnan University, Wuxi 214122, China; lincongyu@jiangnan.edu.cn; 3 Ocean College, Zhejiang University, Zhoushan 316000, China; zhaoshanshan5612@163.com; 4 State Key Laboratory of Urban Water Resources and Environment, Harbin Institute of Technology, Harbin 150090, China

**Keywords:** *Aureobasidium pullulans*, *Cmr1*, melanin, antioxidant activity

## Abstract

We have successfully identified the transcription factor *Cmr1* from the fungus *Aureobasidium pullulans* Hit-lcy3T, which regulates melanin biosynthesis genes. Bioinformatics analysis revealed that the Cmr1 gene encodes a protein of 945 amino acids, containing two Cys_2_His_2_ zinc finger domains and a Zn(II)_2_Cys_6_ binuclear cluster domain located at the N-terminus of *Cmr1*. To investigate the function of the *Cmr1* gene, we performed gene knockout and overexpression experiments. Our results showed that *Cmr1* is a key regulator of melanin synthesis in Hit-lcy3^T^, and its absence caused developmental defects. Conversely, overexpression of *Cmr1* significantly increased the number of chlamydospores in Hit-lcy3^T^ and improved melanin production. RT-qPCR analysis further revealed that overexpression of Cmr1 enhanced the expression of several genes involved in melanin biosynthesis, including *Cmr1*, *PKS*, *SCD1*, and *THR1*. Melanin extracted from the Hit-lcy3^T^ was characterized using UV and IR spectroscopy. Furthermore, we assessed the antioxidant properties of Hit-lcy3^T^ melanin and found that it possesses strong scavenging activity against DPPH**·**, ABTS**·**, and OH**·**, but weaker activity against O_2_^−^**·**. These findings suggest that Hit-lcy3^T^ melanin holds promise for future development as a functional food additive.

## 1. Introduction

Melanin is a complex and diverse large molecule named by the Swiss scientist Jöns Berzelius. It is formed by the polymerization of polyhydroxyindole or polyhydroxyphenolic substances and is widely found in animals, plants, and microorganisms [1]. Currently, melanin can be classified into five types based on the precursor substances of its biosynthetic pathway: L-DOPA (L-3,4-dihydroxyphenylalanine) melanin, 1,8-dihydroxynaphthalene (1,8-DHN) melanin, pyomelanin, pheomelanin, and Glutaminyl-4-hydroxybenzene (GHB) melanin. However, most fungi synthesize 1,8-DHN melanin and L-DOPA melanin via the DHN and L-DOPA pathways, respectively. They form complex polyphenol-based polymers through enzyme-catalyzed and chemical reactions between 1,8-DHN and L-Dopaquinone. Melanin’s amorphous quality renders it insoluble in water, most organic solvents, and acidic solutions, but soluble in alkaline solutions. It is tightly bound to macromolecules such as proteins, carbohydrates, and tannins, making it difficult to accurately analyze its precise structure and chemical composition. Melanin provides protection against UV, chemical, and thermal stresses. It also acts as a potent cation chelator and antioxidant, binds and neutralizes antimicrobial peptides and antibiotics drugs, inhibits enzyme activation, and confers dehydration tolerance [2,3].

Daily foods rich in melanin include coffee, cocoa, dark beer, soy sauce, baked foods, mushrooms, and others [4]. These melanins can provide food with antioxidant capacity, inhibit oxidative stress in cells, suppress or kill pathogenic microorganisms, and induce the synthesis of chemopreventive enzymes [5]. In recent years, melanin’s unique biological characteristics have led to its widespread use in a variety of fields, including food, medicine, cosmetics, and material synthesis [6,7]. Lee et al. found that melanin can effectively reduce the weight of high-fat diet mice, improve lipid status, and increase antioxidant enzyme activity to lower blood lipid levels, and water-soluble melanin complexes have been found to improve insulin sensitivity in obese mice fed a high-fat diet and effectively lower blood sugar [8]. Xu et al. verified that melanin could increase mouse inflammatory factors, alleviate inflammatory stress, and protect the liver [9]. In addition, research has shown that 20% of the total antioxidant capacity intake in the Spanish population is provided by melanin in food [10]. All of these indicate the potential application of melanin as a functional food additive [11]. The characteristics of melanin make it highly valuable, but currently, the process of extracting melanin from animals and plants is complex, with low yield and high cost, making it impossible to obtain melanin in large quantities.

*Aureobasidium pullulans* (*A. pullulans*), commonly known as black yeast, is a fascinating fungus with a complex lifecycle that exhibits various morphological forms under different environmental conditions or growth stages, including budding spores, mycelia, chlamydospores, and small oval-shaped yeast-like cells [12,13]. One of the most notable characteristics of this species is the production of melanin. Researchers have explored different approaches to optimize the production of melanin by *A. pullulans*. For example, Mujdeci used carrot peel extract as a substrate to ferment *A. pullulans* NBRC 100716 and achieved a 100% increase in the total concentration of melanin in the extract by optimizing the fermentation parameters [14]. Mujdeci found that *A. pullulans* NBRC 100716 can also synthesize natural melanin by fermenting food residues, and characterized it after extraction, with the highest yield reaching 300.0 mM/L [15]. Similarly, Gamal et al. achieved a significant increase in melanin production (up to 550.0 mM/L) by optimizing the fermentation parameters using food waste as a substrate [16]. However, most studies on melanin production by *A. pullulans* have focused on the selection of fermentation substrates and strain screening, and further research is needed to better understand the synthesis and regulation of melanin by this species.

*Cmr1* is a crucial transcriptional activator that regulates the melanin synthesis pathway in fungi. It was first discovered by Gento in 2000 and is typically composed of two Cys_2_His_2_ zinc fingers and one Zn(II)_2_Cys_6_ binuclear cluster domain [17]. Since then, homologs of *Cmr1* have been identified in many fungi, including *Helminthosporium maydis*, *Bipolaris oryzae*, *Alternaria brassicicola*, *Alternaria alternate*, *Grifola frondosa*, *Botrytis cinerea*, and *Verticillium dahliae*. These homologs play regulatory roles in the DHN melanin synthesis pathway and affect the virulence of pathogenic fungi [18,19,20,21,22,23,24]. Knockout of the *Cmr1* homolog or downstream regulated genes typically leads to a reduction or loss of melanin production. In the study of melanin production in *A. pullulans*, Jiang et al. initially identified the involvement of the *Cmr1* gene in melanin synthesis but did not conduct further in-depth research on its function [25].

This paper first isolated and characterized the melanin produced by *A. pullulans* Hit-lcy3^T^, and then identified *Cmr1* involved in the synthesis of the melanin through bioinformatics analysis of its whole genome. Subsequently, knockout strains of *Cmr1* were obtained using homologous recombination, and the functions of *Cmr1* were investigated by comparing the morphology, biomass, melanin production, and expression levels of genes involved in melanin biosynthesis between the wild-type (WT), *Cmr1* knockout mutant(*ΔApCmr1*), and *Cmr1* overexpression strains (*OEX-ApCmr1*). Finally, the scavenging activity of the melanin against free radicals including DPPH**·**, ABTS**·**, OH**·**, and O_2_^−^**·** was studied.

## 2. Materials and Methods

### 2.1. Stain, Plasmid, and Culture Medium

*A. pullulans* Hit-lcy3^T^ and DH5α, as well as the PMD-18T vectors and pBARGPE1-EGFP, were obtained from our laboratory. The culture media used in this study included LB (10.0 g tryptone, 10.0 g NaCl, and 5.0 g yeast extract per liter, pH 7.2), YPD (20.0 g/L glucose, 10.0 g/L yeast extract, and 20.0 g/L peptone), seed medium (50.0 g/L sucrose, 2.0 g/L yeast extract, 1.0 g/L NaCl, 6.0 g/L K_2_HPO_4_, 0.2 g/L MgSO_4_, and 0.6 g/L (NH_4_)_2_SO_4_), and fermentation medium (100.0 g/L sucrose, 2.0 g/L yeast extract, 1.0 g/L NaCl, 6.0 g/L K_2_HPO_4_, 0.2 g/L MgSO_4_, and 0.6 g/L (NH_4_)_2_SO_4_).

### 2.2. Acquisition and Bioinformatics Analysis of Cmr1 Gene

The *Cmr1* gene was found in the genome of *A. pullulans* Hit-lcy3^T^, and the primers Cmr1F/Cmr1R were designed to amplify the full-length gene. The amplified gene was sequenced and confirmed by comparison with NCBI (https://www.ncbi.nlm.nih.gov/, accessed on 10 December 2021). SMART (https://smart.embl-heidelberg.de/smart/set_mode.cgi?NORMAL=1, accessed on 5 March 2022) was used to predict the conserved domains of *Cmr1*. BDPG (https://www.fruitfly.org/seq_tools/promoter.html, accessed on 7 March 2022) was used to predict the promoter transcription start site. We then based on Plant CARE (http://bioinformatics.psb.ugent.be/webtools/plantcare/html/, accessed on 25 March 2022) to predict the promoter region of *Cmr1*. ClustalW (https://www.genome.jp/tools-bin/clustalw, accessed on 25 April 2022) and MEGA Ⅹ software were used to perform multiple strain alignment and construct a phylogenetic tree of the amino acid sequence of *Cmr1*.

### 2.3. Melanin Extraction Method

*A. pullulans* Hit-lcy3^T^ was cultured in a fermentation medium for 6, 7, 8, 9, 10, and 11 days, and the biomass was determined. The melanin was then extracted and purified using an acid–ethanol precipitation method. The fermentation broth was thoroughly mixed, and 50 mL of the mixture was centrifuged at 4000 rpm for 10 min. The supernatant was collected for further processing, and the precipitate was the biomass. The collected supernatant was then mixed with 1 mol/L hydrochloric acid to adjust the pH to 2–3 and shaken vigorously. After that, it was left to stand at 60 °C to induce complete precipitation. The product was then filtered through a 600-mesh filter paper and dried at 60 °C to constant weight to obtain the crude melanin. Finally, the crude product was repeatedly dissolved in a strong base, acid–ethanol precipitated and purified to obtain melanin with higher purity.

### 2.4. FT-IR Analysis of Melanin

The preparation method of the FT-IR sample was as follows: the isolated melanin was freeze-dried, ground into powder, and then a suitable amount of the powder was placed in a PerkinElmer 100 FT-IR spectrometer (PerkinElmer Inc., Waltham, MA, USA) for analysis under conditions of a resolution of 4 cm^−1^ and a scanning range of 500–4000 cm^−1^, to obtain the infrared spectrum of the melanin [26].

### 2.5. UV–Vis Analysis of Melanin

A suitable amount of melanin extract and standard substance were dissolved in 1 mol/L sodium hydroxide solution and then placed in a UV-6100 type UV–Vis spectrophotometer to scan the absorption spectrum in the wavelength range of 190–800 nm in spectral scan mode [27].

### 2.6. Plasmid Construction

The primers are listed in Table S1. To construct the *Cmr1* transcription factor knockout plasmid, the PMD-18T plasmid was used as the backbone, and the *Cmr1* gene was replaced with the hygromycin gene using homologous recombination to achieve the deletion of the *Cmr1* gene. First, the Hyg fragment and the upstream and downstream fragments of the *Cmr1* gene (Cmr1-up and Cmr1-down) were amplified by PCR. Then, the PMD-18T plasmid was double-digested with *EcoRI* and *BamHI*, and the Cmr1-up, Hyg, and Cmr1-down fragments were connected to the PMD-18T knockout plasmid backbone using the ClonExpress II one-step cloning kit, thus resulting in the knockout plasmid PMD-Hyg-△Cmr1.

For the construction of the pBARGPE-EGFP-Cmr1 vector, first, a 3013 bp fragment covering the coding sequence of *Cmr*1 was amplified from the genome of *A. pullulans* Hit-lcy3^T^. Then, *EcoRI* and *KpnI* were used to digest the *Cmr1* fragment and pBARGPE1-EGFP vector, respectively. Finally, ClonExpress II one-step cloning was used to ligate the recovered *Cmr1* fragment into pBARGPE1-EGFP, resulting in the overexpression of plasmid pBARGPE-EGFP-Cmr1.

### 2.7. Transformation and Validation of Mutant Strains

The successfully constructed overexpression vector pBARGPE-EGFP-Cmr1 and knockout vector PMD-Hyg-△Cmr1 were transformed into *A. pullulans* protoplasts using the PEG-mediated protoplast transformation method [28]. The genomic DNA of transformants was extracted, and EGFP (egfpF/egfpR) and hygromycin resistance gene (hygR/hygF) were amplified using specific primers and validated by sequencing.

### 2.8. Effect of Cmr1 on Biomass and Melanin Production

Wild-type, *Cmr1* deletion strain, *ΔApCmr1*, and *Cmr1* overexpression strain, *OEX-ApCmr1*, were cultured in a fermentation medium for 9 days to determine their biomass and melanin production.

### 2.9. Morphological Observation

The morphological features of *A. pullulans* Hit-lcy3^T^ and its mutants after 9 days of cultivation on YPD medium were observed using a light microscope (Nikon ECLIPSE E200, Nikon, Tokyo, Japan) and an electron microscope (JSM-6700F, JEOL, Tokyo, Japan). For scanning electron microscopy (SEM) sample preparation, square pieces of 0.5 cm × 0.5 cm were cut from the agar plate, fixed in 2.5% glutaraldehyde at 4 °C for about 12 h, washed twice with phosphate buffer, and treated with graded ethanol (50%, 70%, 90%, 100%). Finally, the samples were placed on conductive tape after treatment with acetone and freeze-dried, and observed under an SEM.

### 2.10. Analysis of the Transcriptional Levels of Genes Related to the ApCmr1 Biosynthetic Gene Cluster

Total RNA was extracted from the WT and different transformants grown in the fermentation medium. EasyScript First-Strand cDNA Synthesis SuperMix (TransGen Biotech, Beijing, China) was used for reverse transcription to obtain cDNA. RT-qPCR was performed using Go Taq qPCR Master Mix (Promega, Madison, Wisconsin, USA) in a real-time PCR system (Thermo Fisher Scientific, Wilmington, Massachusetts, USA), with the β-actin gene as the internal control gene. The relative expression levels of *Cmr1*, *PKS*, *SCD1*, and *THR1* were quantitatively detected, and expression levels were evaluated using the 2^−ΔΔCT^ method.

### 2.11. Investigation of Antioxidant Activity of Melanin In Vitro

#### 2.11.1. DPPH· Scavenging Assay

A 0.2 mmol/L solution of DPPH**·** in absolute ethanol was prepared. Different concentrations of melanin samples and Vc (Vitamin C) solution were mixed with 100 μL of DPPH**·** solution in absolute ethanol, respectively. The mixtures were thoroughly vortexed, and shielded from light. After 30 min. the OD_517_ was measured using a multimode plate reader [12].

DPPH· scavenging rate (%) = [1 − (A_1_ − A_2_)/A_0_] × 100%.

A_0_: 100 μL anhydrous ethanol + 100 μL DPPH**·** absolute ethanol solution;

A_1_: 100 μL test solution + 100 μL DPPH**·** absolute ethanol solution;

A_2_: 100 μL test solution + 100 μL absolute ethanol solution.

#### 2.11.2. ABTS· Scavenging Assay

First, 4 mmol/L ABTS**·** reserve solution and 2.6 mmol/L K_2_S_2_O_8_ solution were prepared. Then, the ABTS**·** reserve solution and the K_2_S_2_O_8_ solution were mixed in a 1:1 ratio and allowed to stand for about 12 h. Finally, it was diluted with distilled water to obtain ABTS**·** working solution with an absorbance of 0.700 at 734 nm wavelength. Next, 100 μL of different concentrations of melanin solution and Vc solution were mixed into 96-well plates, and then 100 μL ABTS**·** working solution was added to each well. After 10 min, the absorbance of the sample at 734 nm wavelength was measured [12].

ABTS**·** scavenging rate (%) = [1 − (A_1_ − A_2_)/A_0_] × 100%

A_0_: 100 μL distilled water + 100 μL ABTS**·**;

A_1_: 100 μL test solution + 100 μL ABTS**·**;

A_2_: 100 μL test solution + 100 μL distilled water.

#### 2.11.3. OH· Scavenging Assay

Then, 1 mL melanin solution and Vc solution of different concentrations were added into 1 mL 9 mmol/L FeSO_4_ and 1 mL H_2_O_2_ in turn. The solution was mixed well and incubated at 37 °C for 10 min. Then, 1 mL 9 mmol/L salicylic acid was added and the reaction continued at 37 °C for 30 min. The absorbance of the sample at 536 nm wavelength was measured [12].

OH**·** scavenging rate (%) = [1 − (A_1_ − A_2_)/A_0_] × 100%.

A_0_: 1 mL distilled water + 1 mL FeSO_4_ + 1 mL H_2_O_2_ + 1 mL salicylic acid;

A_1_: 1 mL of the test solution + 1 mL FeSO_4_ + 1 mL H_2_O_2_ + 1 mL salicylic acid;

A_2_: 1 mL distilled water + 1 mL FeSO_4_ + 1 mL distilled water + 1 mL salicylic acid.

#### 2.11.4. O_2_^−^· Scavenging Assay

In each 6.5 mL reaction mixture, 2 mL L-methionine (39 mmol/L), 2 mL NBT (225 μmol/L), 1 mL disodium EDTA (0.6 mmol/L), 1 mL riboflavin (12 μmol/L), and 0.5 mL of the melanin solution or Vc solution of varying concentrations were added. All solutions were prepared in 0.05 mol/L phosphate buffer (pH 7.8). The reaction mixture containing the sample was incubated at 25 °C for 15 min, and the absorbance was measured at 560 nm. The reaction mixture without the sample served as the control and was kept in the dark. The scavenging activity was calculated as follows [27]:

O_2_^−^**·** scavenging rate (%) = (1 − A_1_/A_0_) × 100%

A_0_: 0.5 mL distilled water + 2 mL L-methionine + 2 mL NBT + 1 mL EDTA + 1 mL riboflavin;

A_1_: 0.5 mL sample solution + 2 mL L-methionine + 2 mL NBT + 1 mL EDTA + 1 mL riboflavin.

### 2.12. Statistical Analysis

All data were statistically analyzed using SPSS 19.0 (IBM Corporation). Gene expression analysis was evaluated using one-way analysis of variance (ANOVA). Duncan’s multiple-range test was used to compare data means. *p* value less than 0.05 was considered statistically significant.

## 3. Results and Discussion

### 3.1. Bioinformatics Analysis of Cmr1

The full length of the *Cmr1* gene in *A. pullulans* is 3013 bp, encoding a protein of 945 amino acids. There are two introns at positions 71 bp–122 bp (50 bp) and 986 bp–1015 bp (30 bp), respectively. SMART analysis of the protein sequence revealed that there are two adjacent Cys_2_His_2_ zinc finger domains located at positions 2–24 bp and 30–52 bp of the amino acid sequence, respectively, and a Zn(II)_2_Cys_6_ binuclear cluster domain located at positions 333–361 bp of the N-terminus of the *Cmr1* protein. The upstream 1500 bp sequence of *Cmr1* was analyzed using the BDPG tool, revealing that *Cmr1* is a member of the GATA family of transcription factors, and its gene promoter region contains three GATA motifs. The cis-acting element analysis of the promoter enhancer region using Plant CARE showed that there are one core promoter element (TATA-box) and three common cis-acting elements (CAAT-box) (Figure S1).

**Figure 1:**
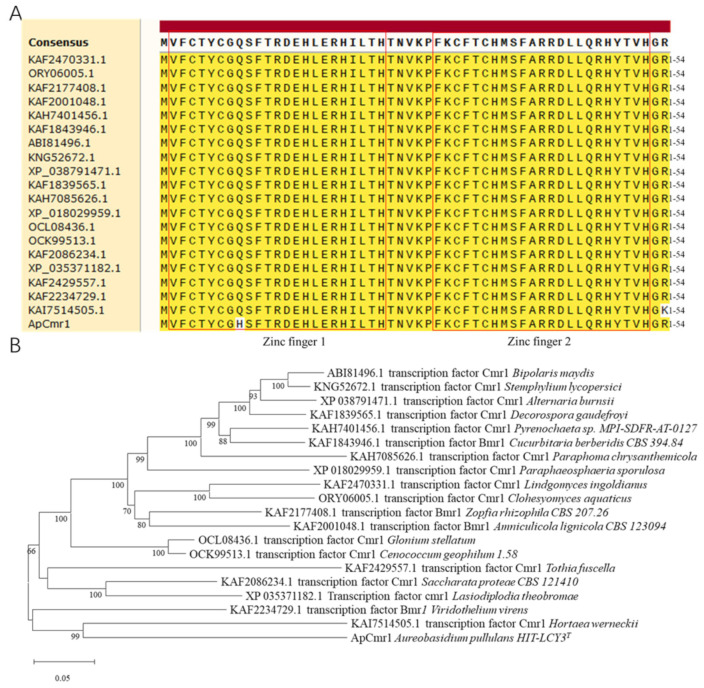
Bioinformatics analysis of *ApCmr1*. (**A**) Comparison of the zinc finger domain of *ApCmr1* and the homologous proteins in 20 fungi. (**B**) Phylogenetic tree of *ApCmr1* and its homologous proteins in 20 fungi.

In the amino acid sequence’s alignment with 20 different fungi, a high similarity was observed in the zinc finger structural sequence of the *Cmr1* gene (Figure 1A). However, *A. pullulans* Hit-lcy3^T^ showed a difference at the 9th amino acid position compared to other strains, with histidine instead of glutamine, which is found in other fungi. Due to its distant evolutionary relationship with *Candida albicans* Ca529L and *Saccharomyces cerevisiae* 218 S288C, it was not included in the phylogenetic tree (Figure 1B).

### 3.2. Fermentation Time of A. pullulans Hit-lcy3T for Melanin Production

**Figure 2:**
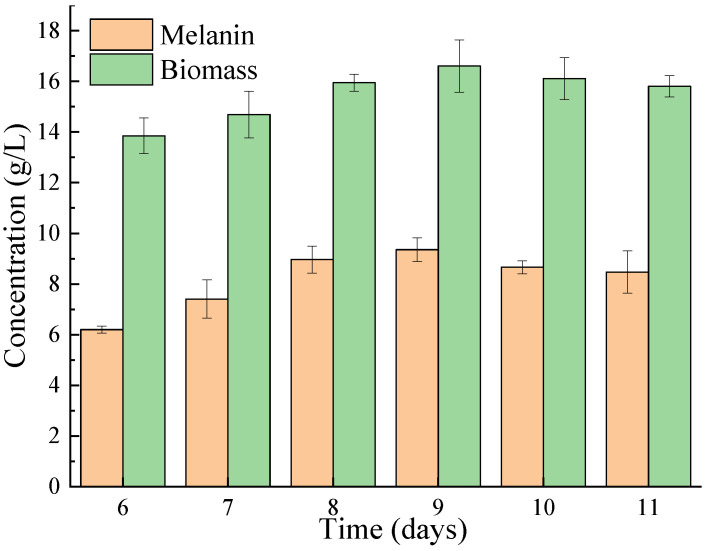
The effect of fermentation time on the biomass and melanin production of *A. pullulans* Hit-lcy3^T^.

The melanin and biomass were measured after different days of fermentation. As shown in Figure 2, both the melanin and biomass increased and then decreased with the increase in fermentation time. At 9 days, the highest melanin yield and biomass were reached at 9.357 g/L and 16.605 g/L, respectively, which were significantly higher than those obtained from *Ophiocordyceps sinensis* and *Streptomyces* sp. (1.775 g/L and 1.46 g/L, respectively) [27,29].

### 3.3. FT-IR Spectroscopy of DHN Melanin

**Figure 3:**
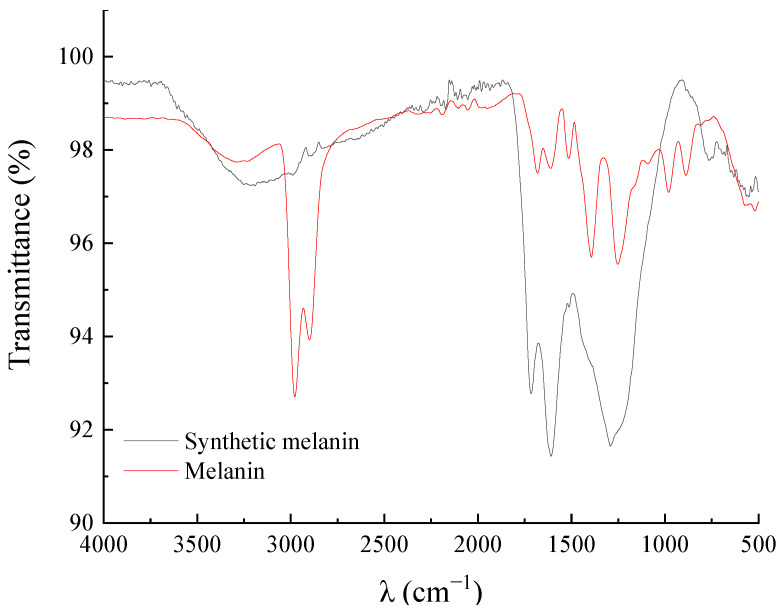
FT-IR of DHN melanin and synthetic melanin.

The obtained melanin was characterized using FT-IR spectroscopy. As shown in Figure 3, the infrared spectrum of the DHN melanin extracted from *A. pullulans* Hit-lcy3^T^ showed similar characteristic peaks as the melanin extracted from *A. pullulans* NBRC 100716, as reported by Mujdeci. The peak at 3300 cm^−1^ is attributed to the stretching vibrations of –OH in aromatic ethers. Two obvious peaks at 2978 cm^−1^ and 2900 cm^−1^ indicate the presence of C–H stretching vibrations in aromatic hydrocarbons. In comparison with the melanin studied by Centeno and Pacelli, which exhibited two obvious peaks at 2950–2850 cm^−1^, the two peaks observed in the present study were attributed to stretching vibrations of C–H in aromatic rings caused by lipids or amino acid residues during the extraction process [26,30]. The characteristic peaks of the melanin and synthetic melanin were the same at 1681 cm^−1^, 1607 cm^−1^, 1521 cm^−1^, 1256 cm^−1^, and 893 cm^−1^. The strong band at 1681 cm^−1^ is attributed to the vibration of C=O [27], while the strong band at 1607 cm^−1^ is attributed to the vibration of the C=C group in aromatic rings [28]. The band at 1521 cm^−1^ is attributed to the N–H bending vibration in the amide group of the aromatic rings and the peak at 1398 cm^−1^ is attributed to the bending vibration of C–H in the aromatic hydrocarbons. The peak at 1256 cm^−1^ is attributed to the stretching vibration of C–N in the amide group of the molecule. The peak observed at 987 cm^−1^ is related to the out-of-plane bending vibration of C–H in the aromatic amine [31].

### 3.4. Analysis of UV–Vis Absorption Spectra of DHN Melanin

**Figure 4:**
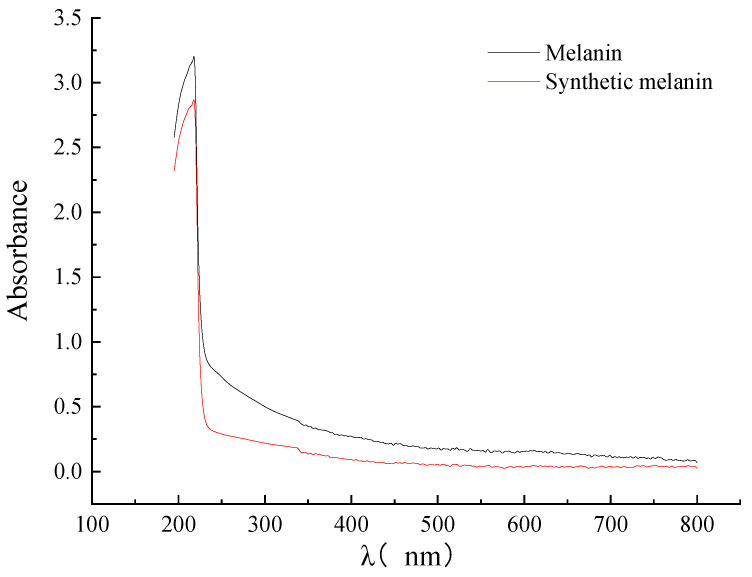
The UV–Vis of DHN melanin extracted from *A. pullulans* and the synthetic melanin.

It has been observed in previous studies that the longer the wavelength, the lower the absorbance of melanin, which is a characteristic feature of melanin. Additionally, the absorption curve of melanin is similar to that of L-DOPA melanin nanoparticles. However, Li et al. found that the absorbance of purified *Streptomyces* sp. melanin showed almost linear behavior and did not exhibit any maximum or minimum absorption within the range of 200–800 nm [27]. Pacelli et al. compared the absorbance spectra of *Cryomyces antarcticus* melanin, L-DOPA, and DHN, and found that the highest peak of absorption was in the range of 200–300 nm [30]. Analysis of the UV–Vis absorption spectra of DHN melanin extracted from *A. pullulans* showed that the maximum absorbance of melanin occurred at 218 nm, which is consistent with the maximum absorbance of synthetic melanin (Figure 4). This is consistent with the results of Thokur’s study on the DHN melanin of *Phyllosticta capitalensis* [32].

### 3.5. Schematic and Verification of Gene Knockout

**Figure 5:**
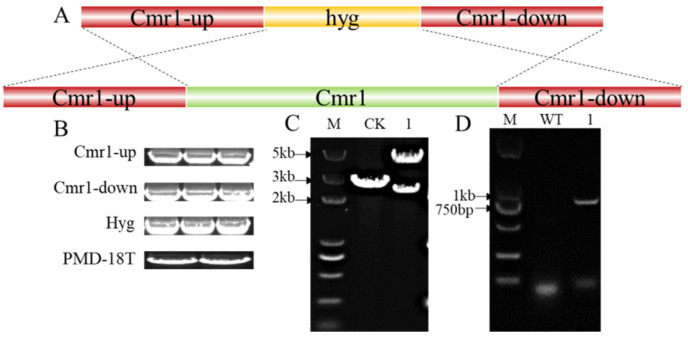
Construction and validation of the *Cmr1* knockout plasmid. (**A**) Construction strategy of the *Cmr1* knockout vector; (**B**) Cmr1-up, Cmr1-down, hyg, and PMD-18T bands after double enzyme digestion; (**C**) enzyme digestion (*EcoRI*) validation of the △Cmr1 vector, where CK represents the PMD-18T vector backbone and 1 represents the △Cmr1 vector; (**D**) PCR validation of the knockout strain, where WT represents the wild-type strain and 1 represents the *ΔAPCmr1* knockout strain.

To verify the gene function of *Cmr1* in *A. pullulans* Hit-lcy3^T^, a knockout vector was constructed using PMD-18T as the vector backbone through homologous recombination, as shown in Figure 5A. Cmr1-up, hyg, Cmr1-down, *HindIII*, and *EcoRI* double enzyme digestion PMD-18T vector bands were validated by agarose gel electrophoresis, with band sizes of 1305 bp, 1412 bp, 1289 bp, and 2635 bp, respectively (Figure 5B). As the △Cmr1 vector contains two *EcoRI* restriction sites while PMD-18T contains only one, *EcoRI* digestion was performed on both PMD-18T and △Cmr1 vectors separately. The resulting bands for △Cmr1 were 2284 bp and 4610 bp, while PMD-18T only produced a band of 2693 bp (Figure 5C). The amplification with the hygR/hygF primers revealed a 1000 bp hygromycin fragment in *ΔAPCmr1*, which was not present in the WT strain (Figure 5D).

### 3.6. Construction and Verification of Cmr1 Overexpression Plasmid

**Figure 6:**
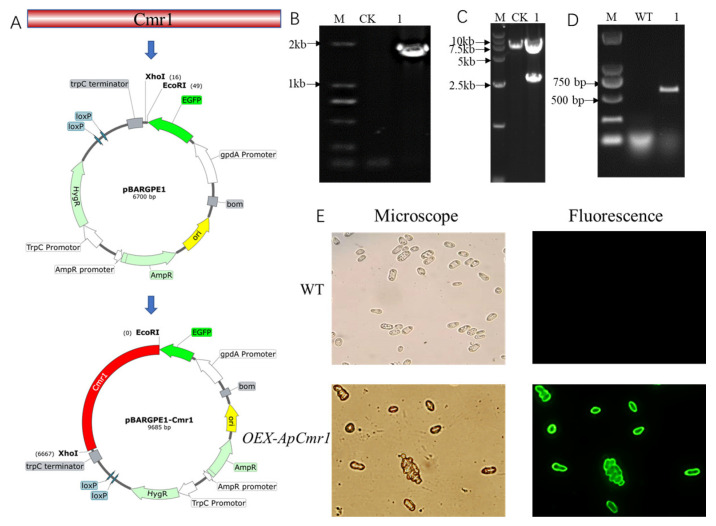
Constructing the *Cmr1* overexpression plasmid and validating the mutant strain. (**A**) Construction strategy for *Cmr1* overexpression vector; (**B**) PCR validation of pBARGPE-EGFP-Cmr1 plasmid, with CK representing the empty vector as negative control and 1 representing pBARGPE-EGFP-Cmr1 plasmid; (**C**) enzyme digestion validation (*BamHI*) of pBARGPE-EGFP vector, with CK representing the empty vector as negative control and 1 representing pBARGPE-EGFP-Cmr1 plasmid; (**D**) PCR validation of the overexpression strain, with WT representing wild-type strain, and 1 representing the *OEX-ApCmr1* overexpression strain; (**E**) green fluorescence validation of the overexpression strain, with WT representing wild-type strain and *OEX-ApCmr1* as the overexpression strain.

Due to the presence of a strong gpdA promoter in pBARGPE-EGFP, it was selected as the backbone for the overexpression vector. The construction process of pBARGPE-EGFP-Cmr1 overexpression plasmid involved double digestion with *EcoRI* and *KpnI*, followed by ligation with the amplified *Cmr1* gene from the genome, as illustrated in Figure 6A. With the pBARGPE-EGFP and pBARGPE-EGFP-Cmr1 as templates, the amplification, using primers epc1-F and epc1-R, showed that a band of 1700 bp was obtained from pBARGPE-EGFP-Cmr1, whereas no band was detected from pBARGPE-EGFP (Figure 6B). Since pBARGPE-EGFP-Cmr1 contains two *BamHI* restriction sites, while pBARGPE-EGFP only has one *BamHI* restriction site, *BamHI* digestion was performed to verify both pBARGPE-EGFP and pBARGPE-EGFP-Cmr1. The results showed that pBARGPE-EGFP-Cmr1 produced two bands of 2672 bp and 6999 bp, respectively, after digestion, while pBARGPE-EGFP produced only a single band of 6700 bp. Furthermore, amplification of the green fluorescent protein-encoding gene using egfpF/egfpR primers revealed a band of 720 bp corresponding to the EGFP gene in the *OEX-ApCmr1* strain but not in the WT strain (Figure 6C). Fluorescence inverted microscope analysis (Figure 6E) confirmed the successful expression of the green fluorescent protein in the *OEX-ApCmr1* strain.

### 3.7. Effect of Cmr1 Mutant Strain on Morphology and Melanin Production

**Figure 7:**
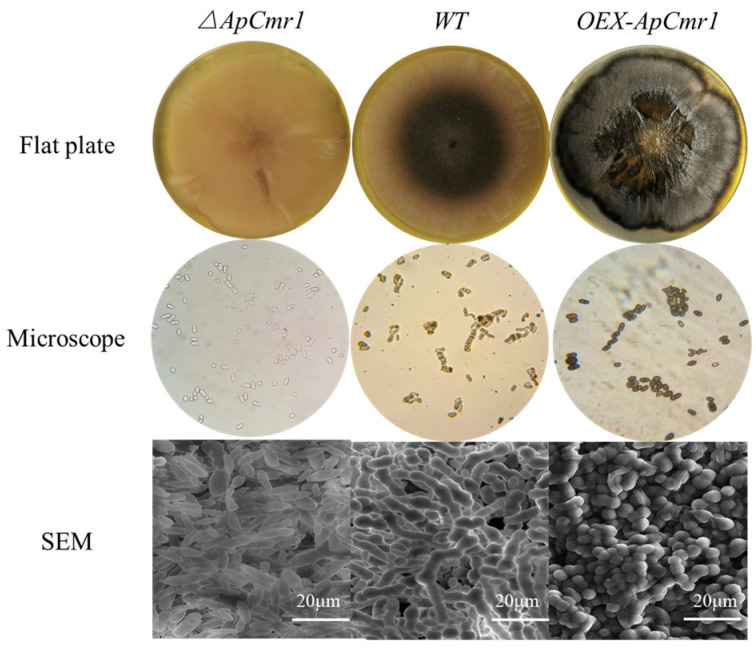
Morphological observation of the mutant strain.

As shown in Figure 7, after 9 days of cultivation in a YPD medium, the surface of the *ΔApCmr1* mutant strain was overall white, smooth, and moist, without aerial mycelia, while both the WT and *OEX-ApCmr1* strains had abundant aerial mycelia on the YPD medium plate. The *OEX-ApCmr1* mutant strain exhibited significantly higher DHN melanin secretion than the WT strain. Under the microscope, there were significant differences in melanin secretion among the two mutant strains and the WT strain, with no melanin generation in the *ΔApCmr1* strain, while the *OEX-ApCmr1* strain had extremely high melanin content, staining the entire cell black. SEM observation revealed that the morphology of *A. pullulans* after knocking out the *ApCmr1* gene was mostly yeast-like, and the yeast cells of the mutant strain had obvious defects in their edges. Overexpression of *ApCmr1* led to chlamydospores becoming the main form, which was consistent with Mujdeci finding that the main form of melanin synthesis is chlamydospores [15]. It is speculated that the GATA-type transcription factor *Cmr1*, as a key transcription activation factor, not only regulates the synthesis of melanin but may also have an effect on the cell morphology of *A. pullulans*.

**Figure 8:**
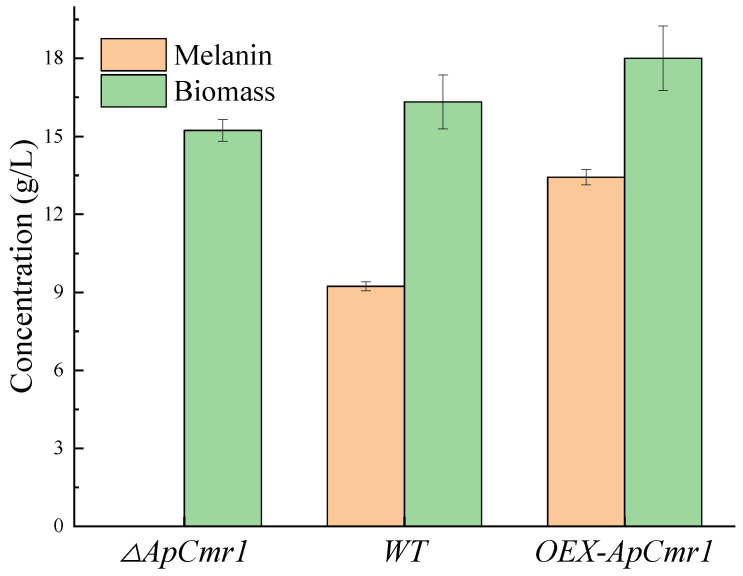
Biomass and melanin production of mutant strains.

WT and each mutant strain were cultured in a fermentation medium for 9 days, and the production of melanin and biomass is shown in Figure 8. The melanin of the *ΔAPCmr1* strain disappeared, and its biomass was reduced compared to the WT and *OEX-APCmr1*. This is partly due to the decrease in the expression level of melanin-related genes and the possible decrease in biomass due to the developmental defects of the strain caused by the knockout of the *Cmr1* gene. However, the melanin production in the *OEX-ApCmr1* strain was 1.436 times that of the WT, and its biomass also slightly increased, which was one of the reasons for the increase in melanin production.

### 3.8. Expression of Melanin Synthesis-Related Gene

**Figure 9:**
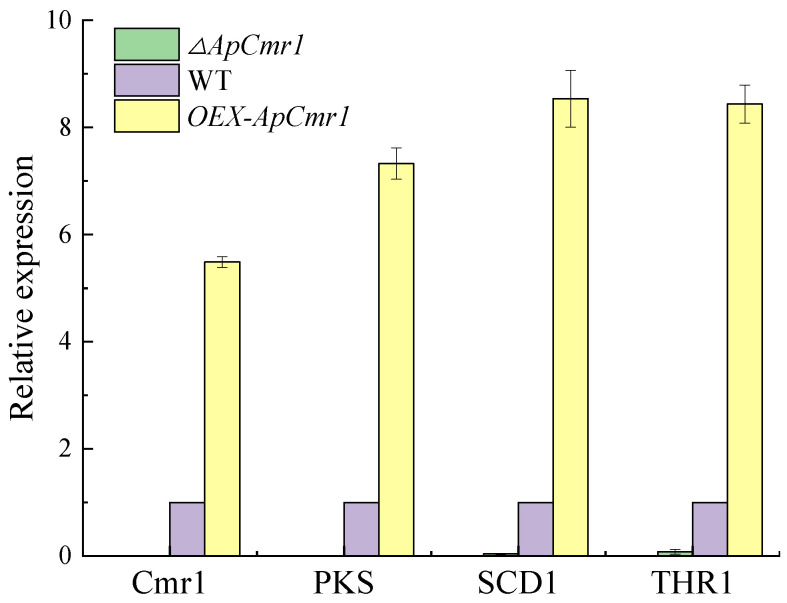
The effect of *Cmr1* on the expression of melanin synthesis genes.

After 9 days of fermentation in the culture medium, RNA was extracted from WT and various mutant strains. Reverse transcription was performed to obtain cDNA, and RT-qPCR analysis was conducted on WT, *∆ApCmr1*, and *OEX-ApCmr1* strains (Figure 9). The results revealed that the expression levels of *Cmr1*, *PKS*, *SCD1*, and *THR1* genes were nearly undetectable after knocking out the *Cmr1* gene, providing further evidence that *Cmr1* acts as a transcriptional regulator of melanin synthesis in *A. pullulans*, which is consistent with the findings of Chi’s study [33]. Overexpression of the *Cmr1* gene resulted in a 5.488-fold increase in the relative expression of *Cmr1*, and 7.324-fold, 8.535-fold, and 8.434-fold increases in the relative expression of *PKS*, *SCD1*, and *THR1* genes, respectively. However, the expression levels were still lower than the relative expression levels of *Bmr1*, *PKS*, *SCD1*, and *THR1* genes in the *Bipolaris oryzae* strain after overexpression of the *Bmr1* gene [19].

### 3.9. In Vitro Antioxidant Activity of Melanin

**Figure 10:**
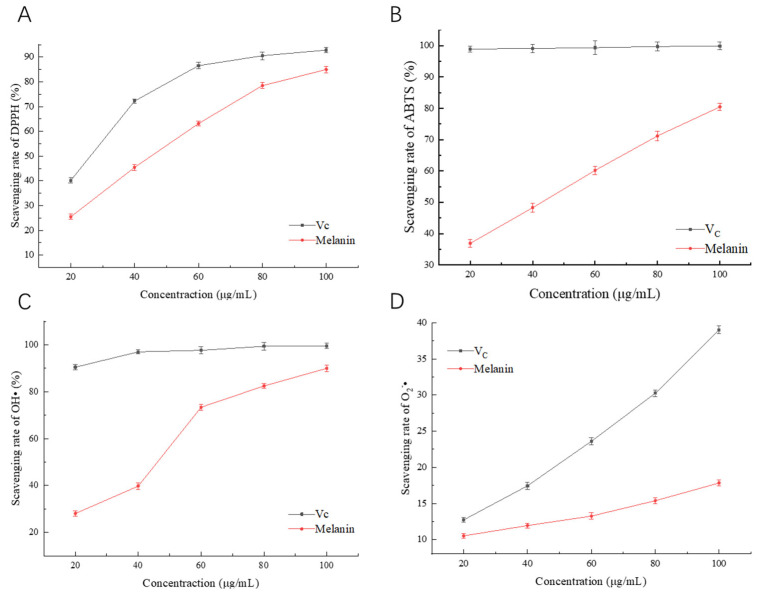
Antioxidant activity of melanin. (**A**) Scavenging rate of DPPH**·**. (**B**) Scavenging rate of ABTS**·**. (**C**) Scavenging rate of OH**·**. (**D**) Scavenging rate of O_2_^−^**·**.

Studies have shown that melanin from microorganisms has certain antioxidant activity. To explore the antioxidant activity of melanin, Vc was selected as a control to evaluate its ability to scavenge DPPH**·**, ABTS**·**, OH**·**, and O_2_^−^**·**. The results of melanin scavenging DPPH**·** are shown in Figure 10A. As the concentration of Vc and melanin increased, the scavenging rate of DPPH**·** also increased, reaching a maximum of 84.96%, which is slightly lower than the ability of melanin extracted from *Cryptococcus rajasthanensis* KY627764 to scavenge DPPH**·** [34]. The melanin scavenging activity against ABTS· is shown in Figure 10B. With the increase in melanin concentration, the scavenging rate of ABTS**·** also increased, reaching a maximum of 80.44%, but it was still lower than that of Vc. The melanin scavenging activity against OH**·** is shown in Figure 10C. With the increase in melanin concentration, the scavenging rate of OH· also increased, reaching a maximum of 81.57%. The ability of melanin to scavenge O_2_^−^**·** increased with the concentration (Figure 10D), reaching a scavenging rate of 17.83% at 100 μg/mL, but was weaker compared to the ability of melanin extracted from *Streptomyces* sp. by Li et al. to scavenge O_2_^−^**·** [27]. These results suggest that the melanin produced by *A. pullulans* has a strong ability to scavenge DPPH**·**, ABTS**·**, and OH**·**, but a weaker ability to scavenge O_2_^−^**·**.

Currently, research on the synthesis and application of melanin is increasing, and the potential of melanin as a new type of functional active substance has been gradually explored. Most fungi synthesize melanin through two pathways: tyrosine (L-DOPA pathway) or malonyl CoA (DHN pathway) [35]. In the DOPA pathway, tyrosinase first catalyzes L-tyrosine (monophenol) and L-DOPA (Bisphenol) to generate L-dopaquinone under the catalysis of the enzyme. Then, L-dopaquinone undergoes enzyme-catalyzed and chemical reactions to form a complex chemical structure, a non-homogeneous polymeric material known as L-DOPA melanin. In the DHN pathway, the precursor of malonyl CoA is endogenously produced. Under the catalysis of polyketide synthase, five malonyl CoA are consecutively decarboxylated and condensed to generate 1,3,6,8-tetrahydroxynaphthalene (THN). THN then undergoes reduction and dehydration to generate 1,8- DHN, which is finally polymerized to obtain DHN melanin. *Cmr1* is a key transcriptional regulatory factor of DHN melanin that regulates the genes involved in the DHN melanin synthesis pathway in most fungi. It specifically binds to the upstream of the polyketide synthase gene to initiate the synthesis of melanin [36]. Studies have shown that in melanin within *A. pullulans*, DHN melanin is the primary form, which is synthesized by the polymerization of 1,8-DHN, and is important for maintaining cell wall integrity and morphology.

Knocking out the *Cmr1* gene caused the disappearance of melanin in *A. pullulans*, as the expression levels of genes related to melanin synthesis were almost zero after knocking out *Cmr1*. Moreover, the biomass of the mutant strains decreased, and they mainly showed a yeast-like morphology. Since fungal melanin is a part of the cell wall composition, the strains that underwent knockout displayed developmental defects around the cell surface, which is consistent with Liang’s study. The reason for this was the loss of melanin, which led to the loss of the originally filled areas of melanin in the cell wall, resulting in irregular developmental defects in the cell wall, demonstrating that melanin plays an important role in maintaining the fungal cell wall morphology [37]. The *OEX-ApCmr1* strain showed increased expression levels of *Cmr1*, *PKS*, *SCD1*, and *THR1* genes related to melanin synthesis by 5.488-fold, 7.324-fold, 8.535-fold, and 8.434-fold, respectively, and melanin production was 1.436-times higher than that of the WT strain. This may be due to the overexpression of melanin in the *OEX-ApCmr1* strain, which bound more to the cell wall and was not released into the fermentation broth. Morphological observations showed that the cells of the *OEX-ApCmr1* strain were predominantly chlamydospores and hyphae, consistent with previous research that suggested that chlamydospores and hyphae were the primary forms of melanin production [38].

The mechanism by which melanin acts as an antioxidant is still unclear. However, recent research has found that its antioxidant activity is related to the monomer 1,8-DHN. This is due to the unique pattern of hydroxylation of the hydrogen-bonding ring in 1,8-DHN, which facilitates the rapid transfer of H atoms [39]. Our research has found that melanin has good scavenging activity on OH**·**, DPPH**·**, and ABTS**·**, but a weak effect on O_2_^−^**·**. The reason for this is that when O_2_^−^**·** extracts H atoms to form OOH· or H_2_O_2_, the transfer of the two hydrogen atoms only occurs at the adjacent carbon position. However, during the polymerization of 1,8-DHN to form melanin, one or two of the adjacent hydroxy groups are replaced, resulting in a weaker scavenging activity on O_2_^−^**·** [40]. In the food industry, natural pigments are valued for their safety, nutrition, and certain bioactivity. Therefore, melanin can be used as a natural coloring agent and additive in food production. Melanin has already been used to prepare yogurt to enhance its antioxidant and free radical scavenging properties, as well as to develop various new types of food, such as black bean vermicelli, black tofu skin, and black caviar [7]. It can also be used as a functional food to better exert its role in reducing blood cholesterol, lowering blood sugar, and anti-tumor activity. There are many microorganisms in nature that can produce pigments. By using genetic engineering methods to obtain genes encoding pigment synthesis and constructing microorganism pigment production engineering bacteria, and effectively regulating their metabolic pathways, it is possible to achieve industrial production of pigments. With the rapid development of biotechnology, microbial pigments are expected to replace synthetic pigments as mainstream food coloring agents.

## 4. Conclusions

In this study, we investigated the regulatory gene *Cmr1* in melanin synthesis through the construction of gene knockout and overexpression strains. Our results showed that *Cmr1* is a key regulator of melanin synthesis in Hit-lcy3^T^, and its absence caused developmental defects. Furthermore, the expression levels of *Cmr1*, *PKS*, *SCD1*, and *THR1* genes related to melanin synthesis were regulated by *Cmr1*. Finally, we found that melanin possesses strong scavenging activity against DPPH**·**, ABTS**·**, and OH**·**, but weaker activity against O_2_^−^**·**. These findings suggest that Hit-lcy3^T^ melanin has the potential to be used as a functional food additive. This study provides a theoretical basis and guidance for the efficient production of melanin strains of *A. pullulans* and research on the antioxidant activity of melanin.

## Data Availability

The data presented in this study are available on request from the corresponding author.

## Supplementary Materials

The following supporting information can be downloaded at: https://www.mdpi.com/article/10.3390/foods12112135/s1, Figure S1: promoter prediction analysis of *ApCmr1*; Table S1: primers used in this study.
