# Supplementary material for: Influence of Cmr1 in the Regulation of Antioxidant Function Melanin Biosynthesis in Aureobasidium pullulans
Source: Foods. 2023 May 25;12(11):2135. doi: 10.3390/foods12112135 (PMC10252820; doi:10.3390/foods12112135)
Supplement: Supplementary file 1 [file foods-12-02135-s001.zip › foods-2375494-supplementary.pdf]

Table S1. Primers used in this study

Primers	Sequence（5’→3’）
Cmr1-F	ATGGTTTTCTGCACATATTGTGGTC
Cmr1-R	TTAGCTTCTCATCAAACGATCAAGT
Cmr1up-F	ATGGTTTTCTGCACATATTGTGGTC
Cmr1up-R	AAGTCGGCTTCATCCCAGG
Cmr1down-F	ACCGAAGCTTGTCGCATTTC
Cmr1down-R	ACAAACCTGTTGGAAGTGCCT
Hyg-F	TCGACAGAAGATGATATTGAAGGAG
Hyg-R	CTATTTCTTTGCCCTCGGACGA
△Cmr1up-F	GTAAAACGACGGCCAGTGCCAAGCTTATGGTTTTCT GCACATATTGTGGTCAC
△Cmr1up-R	CATCTTCTGTCGAAAGTCGGCTTCATCCCAGG
△Hyg-F	TGAAGCCGACTTTCGACAGAAGATGATATTGAAGG AGCAC
△Hyg-R	GACAAGCTTCGGTCTATTTCTTTGCCCTCGGACGAG
△Cmr1down-F	GCAAAGAAATAGACCGAAGCTTGTCGCATTT
△Cmr1down-R	AGCTCGGTACCCGGGGATCCTCTAGAACAAACCTG TTGGAAGTGCCT
OEX-Cmr1-F	TGGACGAGCTGTACAAGTAAGAATTCATGGTTTTCT GCACATATTGTGGTCAC
OEX-Cmr1-R	GTGACGGGCAGGACCGGACGGGGCGGTACCTTAGC TTCTCATCAAACGATCAAGTTTGTTGC
hygF	ATGAAAAAGCCTGAACTCACCG
hygR	CTATTTCTTTGCCCTCGGACGA
epc1-F	GAGTTTGAGGGGATGGTGGG
epc1-R	CCACATGAAGCAGCACGACT
egfpF	TTACTTGTACAGCTCGTCCATGCC
egfpR	ATGGTGAGCAAGGGCGAG
qCmr1-F	GGTCACTCGTTCACCAGGGA
qCmr1-R	TGGAATTAGCCCGAGTTCTGAA
qPKS-F	AAGCCTGAGGAGAAGCTCCAC
qPKS-R	CTGCTGGGGAATGTGGACATT
qSCD1-F	CTTCAACTGGGCAGACAGCTAC
qSCD1-R	TCTAGTGCCACCGATAAAGTGCT

qTHR1-F	TCAAGAAGAACGGTTCCGACG
qTHR1-R	ACGGGTGTTGATCTTGAAGACG

1 GCACTTGGCGACCAACAATCGGCGATCGGGTTTCTTTTGACCTTTACCCCAGAGTTGATGTCGACACGAAACATTAGAGCCTCTTCATTTGACGCCGGAG

CAAT-box

101 CTTTTTGCGACGACATGCCAGTAACAACAACGTTGAGAAAACGGATGTTCATCAACGGTGGAGTGGTGAAAGTGTATCTTTGGACACACCCGATATAGAC

TATA-box

201 TAGTCCAAAGCTTGGCCGGAGATCTCTTGGGAAATCCTTGCACGCCTTGATGAAGGACGACCCCGGCGGCTGAGGCCTGACTAGCAGATCTAGAGTACGC

301 TAGTAAGATATAGTGGCTATGCCGTAAGCACTTCTGAATGGCTCACGGAAGATAAATCTACAGCCCTGTTGGTTGTATATGTTCACCGTGCTACAGGGGC

TATA-box GATA transcription factor binding site TATA-box

401 AGGTACAAGCAAGATCGTCGGATTACTGGATGATGTTGCTCAGGCTGCCCTTGAACGACCAGGTGTCACTGTCGAGGACTGTTTGTACAGAGATCGTCAT

501 TGCGCAGACGCACGAGTGCATCACCATTCCACGATGTATGCTCCGTAAAGTTCCATCGCGTCACGAGAAGGTACAAACGGCTCATGACAGAGTGGGGATT

601 GAGAGAGCTTCCGGGGTGCTGCAGGAAGATCGAGCTGCACGTTAGCTGCTCTGCTGCAAATGCCATGCTAGATCCAATCTCTCCGACTGTCGCTTGGATA

CAAT-box CAAT-box

701 GTGGCTGATCTGGCGCTCCGTCAAAGGCTCGCACGAATTTGATATTTGGCTACGGCTCCGCTGTGCTACCTTAAACGCGATGTGCTTTCCTGACCTGACT

801 TATGCTGACGCCATGATTGTACAGCGCAGGCCTCGACCCGACGACAGAGCAATATCTATATACGGAGTGGACGCGCCGAGCTTGGCGGGCGTATGCCATA

Transcription start site

901 AGCGGCAGCGTTGTGCAGGAAGGTTGACCGTTTTAGGTCTTATCATAGAACACCCGATTGGACTCAAGTCGAACCGCAGTGCCAAGCGCATTCAGATCCG

1001 TCTGCTAGGGTCTTGAAGGTGGGTAGGAGAATTTGGAGGCGGGCTGACTCGTGCATACGTTCGACGATAATGGGGCGGTTGGCGTGCGTCGTAGTGTGTG

GATA transcription factor binding site

1101 TCTCCGACTGGGGCTTATGCTTTGAGCCCGCATGAAACCATACGAAACGTCGCCCATGATAGCTTGATTATGCACCAAATGCGTTCCGCCCCTGGGACCA

GATA transcription factor binding site

1201 TATGCCTCGCTTCGCTGGTACTCTAGTCGACAAGTCATGATGTGTCAAGGATCCGCAACCAGCCCCGAGCTCCATGCTCCTCGCCCAGCAGCTTTTAATA

1301 ATACCATCAGTGTATCCAACTCAGTCAGGCTGGTCCAACACATTAGTAGCACCTCATCTGCTCACCTCTCGCTCTCACTTCTTACCTTTTGTCTCTTTTC

1401 TCCTTCTCATATCTTCTGACCTTCCACATTCTAATCTCCTTCGCTTATCATTGCATGTCCGCGTTACCATTGGCACTCTAATTTACCCCTTCGTCTCACA

Figure S1. Promoter prediction analysis of ApCmr1.
